# Bacteremia detection from complete blood count and differential leukocyte count with machine learning: complementary and competitive with C-reactive protein and procalcitonin tests

**DOI:** 10.1186/s12879-022-07223-7

**Published:** 2022-03-26

**Authors:** Frank Lien, Huang-Shen Lin, You-Ting Wu, Tzong-Shi Chiueh

**Affiliations:** 1 Department of Internal Medicine, Chang Gung Memorial Hospital, Chiayi, Taiwan; 2 Department of Infectious Diseases, Chang Gung Memorial Hospital, Chiayi, Taiwan; 3 Department of Pathology, Chang Gung Memorial Hospital, Chiayi, Taiwan; 4 Department of Laboratory Medicine, Linkou Chang Gung Memorial Hospital, Taoyüan, Taiwan; 5 New Taipei Municipal TuCheng Hospital, TuCheng, New Taipei, Taiwan; 6 Department of Internal Medicine, Chang Gung University, Taoyüan, Taiwan

**Keywords:** Bacteremia, Blood count, Differential count, Machine learning, Procalcitonin

## Abstract

**Background:**

Biomarkers, such as leukocyte count, C-reactive protein (CRP), and procalcitonin (PCT), have been commonly used to predict the occurrence of life-threatening bacteremia and provide prognostic information, given the need for prompt intervention. However, such diagnosis methods require much time and money. Therefore, we propose a method with a high prediction capability using machine learning (ML) models based on complete blood count (CBC) and differential leukocyte count (DC) and compare its performance with traditional CRP or PCT biomarker methods and those of models incorporating CRP or PCT biomarkers.

**Methods:**

We collected 366,586 daily blood culture (BC) results, of which 350,775 (93.2%), 308,803 (82.1%), and 23,912 (6.4%) cases were issued CBC/DC (CBC/DC group), CRP with CBC/DC (CRP&CBC/DC group), and PCT with CBC/DC (PCT&CBC/DC group), respectively. For the ML methods, conventional logistic regression and random forest models were selected, trained, applied, and validated for each group. Fivefold validation and prediction capability were also evaluated and reported.

**Results:**

Overall, the ML methods, such as the random forest model, demonstrated promising performances. When trained with CBC/DC data, it achieved an area under the ROC curve (AUC) of 0.802, which is superior to the prediction conventionally made with CRP/PCT levels (0.699/0.731). Upon evaluating the performance enhanced by incorporating CRP or PCT biomarkers, it reported no substantial AUC increase with the addition of either CRP or PCT to CBC/DC data, which suggests the predicting power and applicability of using only CBC/DC data. Moreover, it showed competitive prognostic capability compared to the PCT test with similar all-cause in-hospital mortality (45.10% vs. 47.40%) and overall median survival time (27 vs. 25 days).

**Conclusions:**

The ML models using only CBC/DC data yielded more accurate bacteremia predictions compared to those by methods using CRP and PCT data and reached similar prognostic performance as by PCT data. Thus, such models are potentially complementary and competitive with traditional CRP and PCT biomarkers for conducting and guiding antibiotic usage.

**Supplementary Information:**

The online version contains supplementary material available at 10.1186/s12879-022-07223-7.

## Background

Bacteremia presents high mortality rates of 22.0–27.7% in health-care and nosocomial settings [1, 2]. To counter this, critical measures, including the prescription of antimicrobics and early diagnosis using various biomarkers, such as leukocyte count, C-reactive protein (CRP), and procalcitonin (PCT), have been adopted into clinical settings for predicting bacteremia before completing a blood culture (BC) [3, 4]. In particular, PCT is well validated for accurately predicting bacteremia, discriminating between contamination and true bacteremia, and even guiding the discontinuation of antimicrobial therapy [5, 6]. It is also capable of determining the prognosis, including the length of hospital stay, mortality [7], and other recovery-related measurements. However, the substantially high cost of PCT testing compared to CRP (USD 39 vs. USD 1) has limited its broader usage [7, 8]. In contrast, despite its lower power (area under the ROC curve [AUC] = 0.569) to predict bacteremia than PCT (AUC = 0.729) [8, 9], CRP remains an effective yet cheaper and more widely available inflammatory marker for guiding antimicrobial therapy. Apart from these biomarkers, recent studies have also shown the ability of machine learning (ML) to predict bacteremia by training prediction models with various clinical data, including vital signs, biochemical and hematologic biomarkers, and so on [9–11]. Although ML models in previous studies have demonstrated promising results, their input data were primarily from various sources that require additional costs and are difficult to obtain in a uniform and timely manner [9–13].

Complete blood count (CBC) and differential leukocyte count (DC) are the two most common laboratory tests applied to patients with unidentified infections. Their turnaround times in the laboratory can be as short as 22 min after implementing the total automation system [14], and the cost per test is about USD 7.00 for CBC and USD 3.50 for DC. Features in CBC/DC have even shown more accurate prediction capability than CRP in the case of bacteremia [3]. Nevertheless, the analysis of CBC/DC data through ML methods, which might provide additional prediction capability instead of using multiple laboratory test results, has not been fully explored.

To fill the gaps, we developed ML models to further utilize readily existing CBC/DC data to determine the occurrence and prognosis of bacteremia using CRP and PCT. Furthermore, we examined whether including CRP or PCT as an additional predictor can improve machine prediction capability. Given that PCT has proven to be more accurate than CRP in determining the prognosis of bacteremia [3], this study excluded CRP when comparing the prognoses of bacteremia. Thus, we formulated the following research questions (RQs).

RQ1: How accurate are predictions of bacteremia made with ML models based on CBC/DC compared to predictions traditionally made using only CRP or PCT levels?
RQ2: Will adding CRP or PCT as a predictor improve the CBC/DC ML model’s prediction capability?
RQ3: How accurate are determinations of bacteremia prognosis regarding all-cause in-hospital mortality and overall median survival time made with ML models based on CBC/DC compared to those made using PCT levels?

## Methods

### Study population

This retrospective study was conducted at Linkou Chang Gung Memorial Hospital (CGMH) in Taiwan, a 3400-bed tertiary medical center that provides care for patients with high degrees of comorbidity. All data used in this study were obtained from Chang Gung Research Database, a duplicate of the CGMH clinical database that was entirely anonymized and delinked [15]. The Institutional Review Board of CGMH (IRB No.: 202001786B0) approved the study and waived informed consent.

**Fig. 1:**
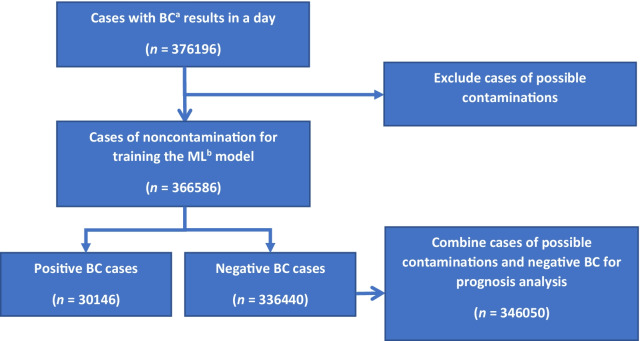
Flowchart of research samples. ^a^Blood culture. ^b^Machine learning

A total of 376,196 consecutive daily BC results were collected (Fig. 1). These were extracted from the records of all adults admitted to CGMH from January 1, 2014, to December 31, 2019, with CBC/DC and BC results concurrently issued on the same day regardless of the order of specimen collection. After excluding 9610 possible contaminations, 366,586 BC cases were obtained. This will be explained in more detail in the “Label cases for ML” section. Of the total number of cases, 350,775 (93.2%) cases issued CBC/DC (CBC/DC group), 308,803 (82.1%) cases issued CRP with CBC/DC (CRP&CBC/DC group), and 23,912 (6.4%) cases issued PCT with CBC/DC (PCT&CBC/DC group) on the same day (Table 1). The patients’ characteristics (age and gender) and yearly case distributions of the three groups were also reported. Cases from 2014 to 2018 were assigned as training and validation sets to build the ML models, whereas those from 2019 were used as testing sets. In investigating RQ3, only 3070 of the 6879 cases were used for prediction because 3809 outpatient cases lacked the prognostic information needed to implement the study.

**Table 1:** The three research groups: CBC/DC^a^, CRP&CBC/DC^b^, and PCT&CBC/DC^c^

Groups	CBC/DC	CRP&CBC/DC	PCT&CBC/DC
Number of cases	**350,775**^d^	**308,803**	**23,912**
Training and validation set	**290,425**	**253,009**	**17,033**
2014	54,729	44,846	2434
2015	55,982	46,775	2898
2016	61,438	53,959	2924
2017	59,843	53,784	4246
2018	58,433	53,645	4531
Testing set	**60,350**	**55,794**	**6879**
2019	60,350	55,794	6879^e^
Age	48.7 ± 30.0	46.8 ± 30.7	53.3 ± 27.6
Male	54.6%	54.3%	58.3%
Female	45.4%	45.7%	41.7%

^a^Complete blood count/differential leukocyte count

^b^C-reactive protein and complete blood count/differential leukocyte count

^c^Procalcitonin and complete blood count/differential leukocyte count

^e^Of the 6879 cases, only 3070 inpatients’ data were used for prediction

^d^Numbers in bold represent the total number of cases

### Training data preparation

A complete CBC/DC test measures several features of the blood, including the following: abnormal lymphocyte, abnormal monocyte, atypical lymphocyte, band, basophil, blast cell, eosinophil, hematocrit, hemoglobin, hypersegmented cell, lymphocyte, mean corpuscular hemoglobin, mean corpuscular hemoglobin concentration, mean corpuscular volume, megakaryocyte, metamyelocyte, monocyte, myelocyte, nucleated red blood cell, platelet, plasma cell, plasmacytoid cell, promonocyte, promyelocyte, red blood cell, red cell distribution width, segmented neutrophil, and leukocyte. Because only a small fraction of patients tested CBC/DC multiple times in 1 day (0.67%; [16]) and minimal intraday variation was expected, the mean result of such multiple, same-day tests was, therefore, used instead of individual test results. For RQ1 and RQ3, each CBC/DC data set was combined into a vector of 28 elements as 1 CBC/DC record in the training set. Regarding RQ2, the patients’ CRP and PCT test values were added to the CBC/DC elements to form the CRP&CBC/DC and PCT&CBC/DC training sets, respectively. Other patient demographic data, such as gender, were excluded in the study, as previous studies have indicated that such data did not significantly affect the probability of bacteremia [9, 13].

### Label cases for ML

Bacteremia refers to the recovery of any bacteria in at least one BC set. To avoid mislabeling data and consequently reducing the ML model’s performance, the recovered coagulase-negative staphylococci, diphtheroids, *Micrococcus* sp., and *Bacillus* sp. were considered to be contaminated and were excluded in establishing and training the predicting model [4]. Only cases of noncontamination with positive recovery were labeled as positive cases (*n* = 30,146) for the ML training model (Fig. 1). However, all excluded cases (*n* = 9610) were merged with the negative culture cases (*n* = 336,440) to analyze RQ3 further.

### ML methods

We included the logistic regression method into our model comparison following its frequent usage in multivariate analyses. Moreover, the random forest model has been shown to readily differentiate the importance of features and obtain a satisfying AUC [9, 16, 17]. However, considering that other ML methods, such as the artificial neural network (ANN), showed a mixed comparative performance compared to the logistic regression and random forest models [18], we also compared their performance using the CBC/DC group to justify the ML method selection.

### Model training, validation, and testing

The training and validation sets for building ML models based on CBC/DC in RQ1 included data from 290,425 cases collected from 2014 to 2018 (Table 1). For RQ2, in comparing the capability of the ML models obtained with and without a biomarker (i.e., CRP), both ML models were trained and validated with 253,009 cases. Similarly, in comparing the addition of PCT as a predictor, 17,033 cases were used for training and validating both ML models. In line with RQ3, the better ML model obtained in RQ1 was used. Fivefold cross-validation was used for all ML methods, and the last validated model was saved to make predictions in this study. Data were split annually, and models were trained over 4 years and tested over the 5th year. Data from 2019 on 60,350 cases served as testing sets for RQ1; 55,794 cases, for RQ2; and 3070 cases, for RQ3.

### Performance evaluation

RQ1 was addressed by measuring the AUC to represent the ML models’ capability. In response to RQ2, aside from evaluating AUCs, the Pearson correlation coefficient was used to test the correlation between models with and without CRP or PCT as predictors.

As for RQ3, Youden’s index [19] was calculated to select the optimal cutoff value for ML models and biomarkers in the last cross-validation. Sensitivity, specificity, positive predictive value (PPV), negative predictive value (NPV), positive likelihood ratio (LR+), and negative likelihood ratio (LR−) were also calculated to evaluate the performance of the selected optimal cutoff on the testing set. The last validated ML model based on CBC/DC data was used to predict each case in the testing set of the PCT&CBC/DC group. Furthermore, positive cases were defined by the selected optimal cutoff value, and their survival curve was compared to that of the negative cases through the Kaplan–Meier survival analysis and log-rank test. Overall median survival time and all-cause mortality were also documented and compared.

Meanwhile, initially excluded cases of possible contaminations were merged with BC negative cases and then evaluated with the last validated model and PCT value in the same manner as the noncontaminated data previously described. Because the AUC, sensitivity, and specificity were relatively unreliable in these possibly contaminated cases, the trained model would be evaluated in terms of the accuracy of prognosis. The importance of each feature in the random forest model was then plotted to depict the model’s behavior and provide implications for clinical judgment. Coefficients of logistic regressions were also calculated for comparisons.

As previous studies have mainly focused on using the lens of the ROC curve [5, 20] in comparing the ML model performance with past literature, this study will thus retain reporting its AUC. For imbalanced data, the Precision-Recall AUC (PRAUC) has been shown to provide more information given its focus on the tradeoff between sensitivity and PPV [21]. Thus, we also provided the PRAUC results of the comparisons between random forest models and PCT for a better understanding of their comparative performance.

All ML models and calculations, including the AUC, Youden’s index, Kaplan–Meier survival analysis, log-rank test, and other metrics, were performed using the “sklearn,” “statsmodels,” “lifelines,” and “kaplanmeier” [22] packages in Python 3.4 (https://www.python.org/). In addition, statistical analyses of variance and the correlation coefficient were executed in SPSS (IBM SPSS Statistics 19, Chicago, IL).

## Results

In addressing RQ1, the bacteremia prediction capability of ML models and those predictions made by different biomarkers (CRP or PCT) were measured through cross-validation and testing data, and the results of the AUC were presented (Table 2). The random forest model trained with CBC/DC showed satisfactory performance in predicting bacteremia in the CBC/DC group with an AUC of 0.802 (Table 2; the ROC graph is also shown in Additional file 3: Figure S1.a). Then, the model was compared to CRP and PCT in terms of bacteremia prediction for the CRP&CBC/DC group and PCT&CBC/DC group, respectively. While the random forest model obtained an AUC of 0.806 and 0.767 on the two groups, the predictions made by traditional biomarkers alone only achieved an AUC of 0.699 and 0.731, respectively. The ROC graphs that use PCT as their only marker for predicting bacteremia in the PCT&CBC/DC group compared to that of the random forest model trained solely with CBC/DC for predicting bacteremia in the PCT&CBC/DC group are shown (see Additional file 3: Figs. S2a and S3a). The evidence supports the competitive performance of random forest models based on CBC/DC data compared to that based on traditional CRP or PCT biomarkers.

**Table 2:** Bacteremia prediction capability indicated with AUCs^a^ of biomarkers (CRP/PCT) and models (random forest/logistic regression)

Methods/Group	CBC/DC^b^		CRP&CBC/DC^c^			PCT&CBC/DC^d^		
	Cross-validation	Testing		Cross-validation	Testing		Cross-validation	Testing
Used biomarker	–	–	CRP	0.692 ± 0.017	0.699	PCT	0.748 ± 0.021	0.731
ML^e^ models								
Random forest	0.792 ± 0.010	0.802	CRP excluded^f^	0.797 ± 0.010	0.806	PCT excluded^h^	0.759 ± 0.022	0.767
			Included^g^	0.806 ± 0.011	0.814	Included	0.777 ± 0.018	0.767
Logistic regression	0.763 ± 0.009	0.772	Excluded	0.769 ± 0.009	0.775	Excluded	0.735 ± 0.030	0.734
			Included	0.784 ± 0.011	0.790	Included	0.761 ± 0.024	0.745

^a^Areas under the ROC curve

^b^Complete blood count/differential leukocyte count

^c^C-reactive protein and complete blood count/differential leukocyte count

^d^Procalcitonin and complete blood count/differential leukocyte count

^e^Machine learning

^f^Trained and validated based on CBC/DC data of CRP&CBC/DC group (n = 253,009)

^g^Trained and validated based on CBC/DC and CRP data of CRP&CBC/DC group

^h^Trained and validated based on CBC/DC data of PCT&CBC/DC group (n = 17,033)

As a response to RQ2, the random forest model using only CBC/DC data excluded CRP data from the CRP&CBC/DC group, obtaining an AUC of 0.806 for the testing set. Adding CRP as a predictor to CBC/DC only slightly enhanced its bacteremia prediction capability (AUC = 0.814). In contrast, adding PCT as a predictor to CBC/DC did not change its prediction capability (AUC = 0.767). Similar to what was found in RQ1, the random forest model performed better than the logistic regression model in both the CRP&CBC/DC and PCT&CBC/DC groups. It also showed a similar result in which adding a biomarker (CRP or PCT) did not significantly change prediction capability.

The model trained using only CBC/DC data was then used to evaluate performance metrics, and Youden’s index optimal cutoff was determined from the last cross-validation model, as previously stated. Optimal cutoffs of CRP and PCT were also calculated for the CRP&CBC/DC and PCT&CBC/DC groups using the last cross-validating data. Other evaluation metrics were calculated as well. The random forest model and CRP biomarker method (cutoff = 46.1 mg/L) used on CRP&CBC/DC group testing data had the following values: sensitivity (75.0% vs. 63.9%), specificity (72.3% vs. 63.9%), PPV (20.0% vs. 14.0%), NPV (96.9% vs. 95.1%), LR+ (2.70 vs. 1.77), and LR− (0.35 vs. 0.56), respectively. These results demonstrate the superior performance of the random forest model. Meanwhile, in the PCT&CBC/DC group, the random forest model also displayed overall superior performance in sensitivity (82.8% vs. 74.8%), specificity (55.8% vs. 59.5%), PPV (20.2% vs. 20.0%), NPV (96.0% vs. 94.6%), LR+ (1.87 vs. 1.85), and LR− (0.31 vs. 0.42) compared to PCT (cutoff = 0.47 ng/mL) on PCT group testing data. These metrics have consistently indicated that ML methods outperform biomarkers (CRP or PCT).

**Fig. 2:**
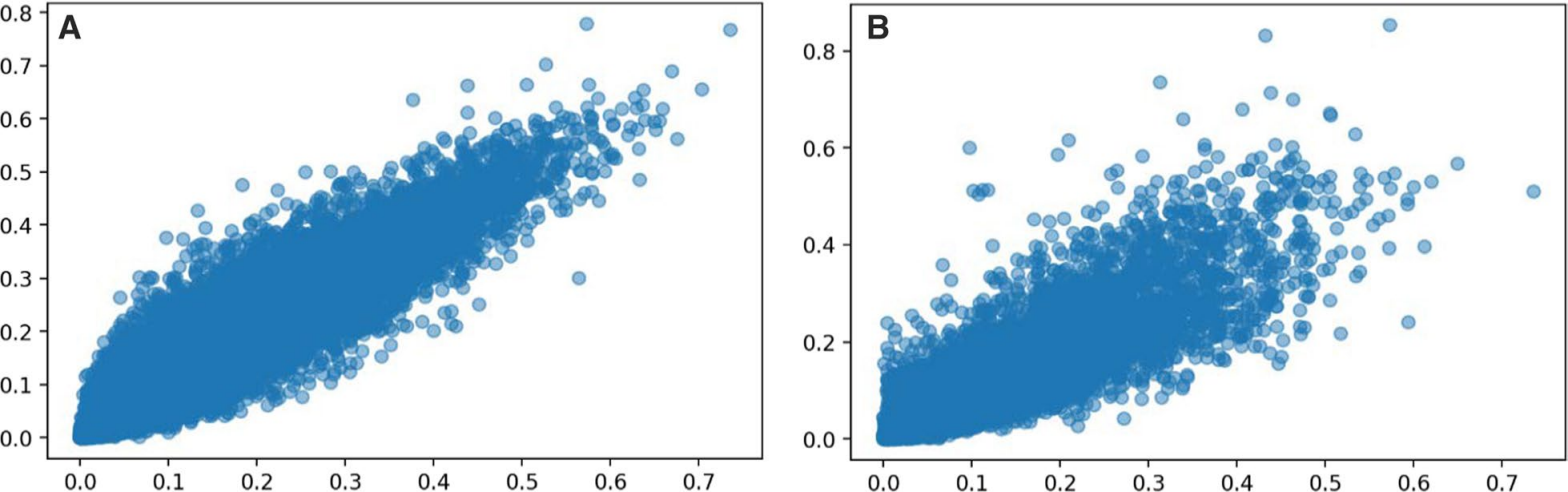
Testing set correlation between models. (**A**: excluding/including CRP^a ^, *r* = 0.851,* p* < 0.005; **B**: excluding/including PCT^b^. (*r* = 0.851, *p* < 0.005); (, *r* = 0.951, *p* < 0.005). ^a^C-reactive protein. ^b^Procalcitonin

Concerning RQ1 and RQ2, the results demonstrated that the random forest model possesses a better prediction capability than logistic regression, which is consistent with previous publications where random forest models outperformed logistic regression models. Thus, our study mainly focused on the random forest model. To reveal the correlations of prediction capability when excluding or including a biomarker in the predicting model, we further tested and obtained a high correlation (*r* = 0.851, *p* < 0.005, strong positive correlation) between the CBC/DC random forest model and CRP&CBC/DC random forest model in predicting PCT&CBC/DC testing sets. Similar prediction results were obtained from the CRP&CBC/DC group (*r* = 0.951, *p* < 0.005, very strong positive correlation) [23]. Detailed correlation plots between (A) models excluding CRP vs. including CRP and (B) models excluding PCT vs. including PCT were then shown (Fig. 2).

Regarding RQ3, the prognostic information of the positive and negative groups, determined by optimal cutoff values, was summarized (Table 3). All-cause in-hospital mortality and overall median survival time were applied as prognostic information. The last model provided a similar prognostic performance with PCT alone in all-cause in-hospital mortality (45.10% vs. 47.40% for the positive group and 14.50% vs. 19.40% for the negative group) and overall median survival time (27 vs. 25 days for the positive group and 58 vs. 58 days for the negative group). Prognostic performance still showed similar results even in evaluating the model’s performance in cases of possible contaminations. The Kaplan–Meier survival analysis and log-rank test also demonstrated a significant difference over four risk groups (*p* < 0.005) for the last model (random forest) and through prediction with PCT level alone for both sets of the pathogenic bacteria isolates group and contaminations group.

**Table 3:** Comparative prediction capability for prognosis through PCT^a^ level and random forest model based on CBC/DC^b^

Blood cultures	Prediction methods			
	Random forest		PCT level	
With pathogenic bacterial isolates				
Group	Positive	Negative	Positive	Negative
N	1888	1098	1536	1450
All-cause in-hospital mortality	45.10%	14.50%	47.40%	19.40%
Overall median survival (days)	27	58	25	58
With possible contaminations				
Group	Positive	Negative	Positive	Negative
N	1604	1067	1285	1386
All-cause in-hospital mortality	42.50%	14.40%	44.50%	19.00%
Overall median survival (days)	29	58	28	58

^a^Procalcitonin

^b^Complete blood count/differential leukocyte count

**Fig. 3:**
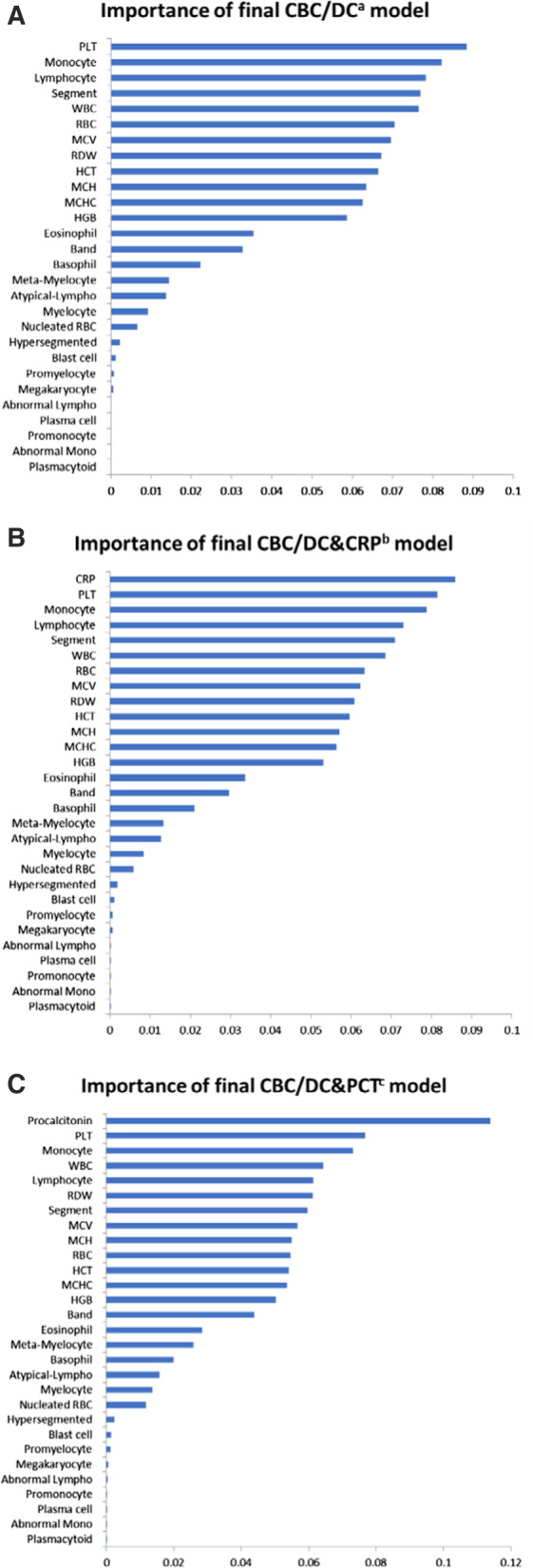
Feature importance of final **A** CBC/DC^a^, **B** CBC/DC&CRP^b^, and **C** CBC/DC&PCT^c^ models. ^a^Complete blood count/differential leukocyte count. ^b^Complete blood count/differential leukocyte count and C-reactive protein. ^c^Complete blood count/differential leukocyte count and procalcitonin

Feature importance in the latest random forest models, models trained from the CBC/DC set within the CBC/DC group, models trained from additional CRP data within the CRP group, and models from additional PCT data within the PCT group were all plotted (Fig. 3). Although CRP and PCT provided the highest contribution compared to other features, CRP only provided importance slightly higher than platelet count and monocyte percentage. From our CBC model, we found that platelet count, monocyte percentage, lymphocyte percentage, segmented neutrophil percentage, and leukocyte count ranked top five in feature importance, whereas features ranked 6th–12th were all those related to the red blood cell series. The average feature importance was 0.0357. Twelve features had relatively higher importance, which dropped drastically in those after the 12th rank. Coefficients, *p*-values, and confidence intervals in the logistic regression analysis were also shown (see Additional file 1: Table S1).

## Discussion

We demonstrated a desirable performance in determining the occurrence and prognosis for evaluating bacteremia using ML analysis with solely CBC/DC data, even without PCT. The results implied the applicability of ML models for predicting bacteremia in daily clinical practice. In addressing RQ1, we found that the random forest model trained with CBC/DC data achieved an AUC of 0.802, which is superior to predictions made with CRP or PCT levels. As a response to RQ2, we found that the AUC has not substantially increased upon the addition of either CRP or PCT to CBC/DC data compared to the ML models purely based on CBC/DC data. Moreover, the detailed correlation plots between (A) models excluding vs. including CRP and (B) models excluding vs. including PCT also showed very strong and strong correlations with a high significance of Pearson correlation coefficients, respectively. Therefore, the predictive model trained with only CBC/DC data can be inferred to have provided a similar level of prediction capability on the occurrence of bacteremia without using any CRP and PCT test results as predictors. As for RQ3, in the PCT&CBC/DC group, our ML prediction has shown competitive capability in determining prognosis for PCT. Building on previous studies, which have substantiated the role of CRP and PCT in facilitating antimicrobial therapy [7, 8], the results allow us to conclude that ML models using solely CBC/DC data may substitute or enhance both biomarkers for guiding antibiotic usage.

The results used in answering RQ1 demonstrated the value of the study’s ML method. Previously, two systematic reviews were conducted in 2015, reassuring the prediction performance of PCT in sepsis/bacteremia [5, 20] and reported an overall ROC (0.79 and 0.77), sensitivity (76% and 76%), and specificity (69% and 64%), which are consistent with the predicting power of PCT in our study (ROC = 0.731, sensitivity = 74.8%, and specificity = 59.5%). The most widely used and optimal cutoff of 0.5 ng/mL in one study is also almost identical to our PCT results calculated from Youden’s index (0.47 ng/mL) [5]. While the diagnostic capability is consistent with those studies, our random forest model trained solely from CBC/DC data (ROC = 0.759, sensitivity = 82.8%, and specificity = 55.8%) provides both higher LR+ and lower LR− while comparing them with PCT in the same group of patients. This indicates that our study’s overall predicting capability is higher, as it identifies patients with and without suspected bacteremia. In addition, a PPV of 20.2% and NPV of 96.0% were obtained, which are both superior to PCT. This further demonstrates the strong potential of implementing the model into clinical scenarios where excluding bacteremia is needed.

Notably, the comparative performance of the ANN, random forest, and logistic regression models (see Additional file 2: Table S2) showed that the ANN demonstrated a similar performance as random forest. The random forest and logistic regression models both showed relative consistency in the final cutoff value, providing the variable’s importance/influence level to some extent. Hence, the inclusion of random forest and logistic regression models in our study is justified.

As mentioned previously, the PRAUC provides a closer look into the prediction performance of imbalanced data. The performance of our random forest model (see Additional file 3: Fig. S3b) has also demonstrated that it outperformed the prediction made using PCT as the only marker (Additional file 3: Fig. S2b) in each of the cross-validation and testing sets (e.g., 0.320 vs. 0.315 in 2014, 0.376 vs. 0.328 in 2015, etc. Data shown in Additional file 3: Figs. S2b and S3b). The results of PCT’s PRAUC are also consistent with the previous study, which provided the sensitivity and PPV of different cutoff levels of PCT for the diagnosis of bacteremia in acute fever settings [24]. These results (Additional file 3: Figs. S1a–S3b) prove the robustness of our model performances.

As stated in the “Training data preparation” section, the ML methods in this study are mainly trained from a vector of 28 elements obtained from patients’ complete CBC/DC tests, of which no other biochemistry data, such as sodium, potassium, blood urea nitrogen, creatinine, and alanine aminotransferase, were used for the analysis. The reasons are twofold: first, the percentages of missing biochemistry data in current hospital testing settings (see Additional file 2: Table S3) would cause a problem in building a functional prediction model. Second, even for retained cases with those biochemistry data, the ML models developed by including additional biochemistry data showed a decreased performance compared to ML models developed in this study based on CBC/DC (see Additional file 2: Table S4). As incorporating biochemistry data decreases model performance and limits overall usage compared to adopting solely CBC/DC, abandoning biochemistry data in our final analysis leads us to establish more applicable and reliable prediction models.

Although the BC results can be unreliable in groups of possible contaminations, our model still obtained similar effective prognostic performance as that with PCT. As previous studies successfully discriminated between bacteremia and contamination with PCT levels [5, 6], this study’s model may provide a similar quality of information to avoid unnecessary antimicrobial usage and decrease microorganism selection pressure. With these benefits, related expenditures can be reduced, as false-positive results may increase the length of one’s hospital stay by 4.5 days and total charge by USD 6878 per case [25, 26].

Regarding our study’s medical resources and financial aspect, CRP and PCT were ordered 60,350 and 6879 times in 2019 and cost approximately USD 60,350 and USD 268,281, respectively [7]. Despite physicians’ judicious and careful selection of cases in current practice, while most of the cases are tested with CRP, PCT tests and BCs do not indicate bacteremia [27–29]. Our model, in particular, is more accessible given the widespread implementation of CBC/DC tests in clinical settings, whereas CRP and PCT frequently need additional blood drawn, labor, and testing expenses. Furthermore, our model can be executed in a timely manner because of the computing power nowadays (about 0.05 s per case on a personal computer), and it can provide results almost simultaneously following the CBC/DC report. This provides the opportunity to steward antimicrobial therapy earlier and avoid unnecessary antimicrobial usage compared to the current CRP- and PCT-guided practices. A previous study has also confirmed that both CRP- and PCT-guided practices provide comparable performances from aspects of mortality, length of hospital stay, and antibiotics usage [7]. PCT-guided practices certainly save overall costs after the expense of relatively small additional costs on PCT tests themselves [8]. In comparison, our ML approach could have greater savings on overall cost as clinicians may then initiate an infection workup and consider early antimicrobial therapy if identified as a high risk of bacteremia and discontinue unnecessary antibiotics more promptly and accurately after a comprehensive evaluation of the patient’s clinical condition.

After depicting feature importance in the models, the five most vital features were platelet count, monocyte percentage, leukocyte count, segmented neutrophil percentage, and lymphocyte percentage. This finding is consistent with previous studies regarding infectious markers and recent studies that emphasize the monocyte as an indicator of infection [30–32]. Our study included all red blood cell indexes and analyzed their importance in ML models, whereas previous studies only depicted a few selected items [13]. Conventionally, leukocyte count is often considered an essential marker of infectious diseases. However, our model has shown that both platelet count and monocyte percentage are even more important than leukocyte count in diagnosing bacteremia [33].

Recent ML studies have exhibited promising results in predicting bacteremia from multiple sources of training data, such as vital signs, sequential organ failure assessment scores, biochemical and hematologic biomarkers, and so on [9–13]. A study in 2018 also concluded that an ML method trained with data consisting of biochemical and hematologic biomarkers and cytokines is unable to improve the diagnostic accuracy of PCT [9]. However, our study presented the first ML method based solely on CBC/DC data and trained with a big set of data, which is feasible with only one blood draw and one hematology analyzer. With this, a short turnaround time of about 22 min in clinical settings can be achieved [14].

Although our study provided a potentially powerful approach to improving bacteremia prediction, it has several limitations. First, because it is a retrospective single-center study, the patient population might gradually shift once the model is implemented into clinical practice. In cases wherein training data are obtained from patients tested with CBC/DC, CRP, and PCT after the physician’s clinical judgment, training data might affect the final performance in different organizations. However, our ML approach demonstrated robustness and a desirable AUC across the CBC/DC, CRP&CBC/DC, and PCT&CBC/DC groups. The results also indicated that our model could still perform well even in different patient populations. Second, our study was only limited to investigating bacterial bloodstream infections. Third, our study focused only on laboratory data and did not incorporate any patient’s underlying conditions and comorbidities. Further study implementing patient condition stratification may provide a more promising result. Fourth, it was conducted before the COVID-19 pandemic, so the model’s performance under specific pandemic settings has yet to be explored. Fifth and last, it only evaluated the prognostic performance in terms of in-hospital mortality rate and overall median survival time. Future randomized, prospective studies may be needed to confirm this ML approach’s performance in the stewardship of antimicrobial usage and identification of contamination.

## Conclusions

In summary, this study has demonstrated that ML approaches using solely CBC/DC data may better predict the occurrence of bacteremia and determine its prognosis similar to PCT and could potentially discriminate contamination from true bacteremia. Both advantages have shown the models’ high potential as a complementary and competitive method for CRP and PCT tests once implemented in clinical settings.

## Supplementary Information

**Additional file 1: Table S1.** Coefficients, p-values, and confidence intervals in logistic regression analysis. This shows data reran using logistic regression in Python’s statsmodels package.

**Additional file 2: Table S2.** The AUC performance of different methods with solely CBC/DC data. **Table S3.** Missing data percentages of biochemistry panels. **Table S4.** Incorporating biochemistry data compared to CBC/DC only in cases without missing values using random forest.

**Additional file 3: Figure S1.** **a** AUC performance of random forest model trained with CBC/DC for predicting infection in CBC/DC group. **b** PRAUC performance of random forest model trained with CBC/DC for predicting infection in CBC/DC group. **Figure S2.** **a** AUC performance of using procalcitonin as the only marker for predicting infection in PCT&CBC/DC group. **b** PRAUC performance of using procalcitonin as the only marker for predicting infection in PCT&CBC/DC group. **Figure S3.** **a** AUC performance of random forest model trained with CBC/DC for predicting infection in PCT&CBC/DC group. **b** PRAUC performance of random forest model trained with CBC/DC for predicting infection in PCT&CBC/DC group.

## Data Availability

Data that support the findings of this study are available from Chang Gung Research Database, Chang Gung Memorial Hospital. However, restrictions apply to the availability of these data, which were used under license for the current study and so are not publicly available. Still, such data are available from the authors upon reasonable request and with the permission of Chang Gung Research Database, Chang Gung Memorial Hospital.

## Abbreviations

ANN: Artificial neural network
AUC: Area under the ROC curve
BC: Blood culture
CBC/DC: Complete blood count/differential leukocyte count
CGMH: Chang Gung Memorial Hospital
CRP: C-reactive protein
LR+: Positive likelihood ratio
LR−: Negative likelihood ratio
ML: Machine learning
NPV: Negative predictive value
PCT: Procalcitonin
PRAUC: Precision-Recall AUC
PPV: Positive predictive value
ROC: Receiver operating characteristics
RQ: Research question

## References

[1] Søgaard M, Nørgaard M, Dethlefsen C, Schønheyder HC (2011). Temporal changes in the incidence and 30-day mortality associated with bacteremia in hospitalized patients from 1992 through 2006: a population-based cohort study. Clin Infect Dis.

[2] Kumar A, Roberts D, Wood KE, Light B, Parrillo JE, Sharma S (2006). Duration of hypotension before initiation of effective antimicrobial therapy is the critical determinant of survival in human septic shock. Crit Care Med.

[3] de Jager CPC, van Wijk PTL, Mathoera RB, de Jongh-Leuvenink J, van der Poll T, Wever PC (2010). Lymphocytopenia and neutrophil-lymphocyte count ratio predict bacteremia better than conventional infection markers in an emergency care unit. Crit Care.

[4] Riedel S, Melendez JH, An AT, Rosenbaum JE, Zenilman JM (2011). Procalcitonin as a marker for the detection of bacteremia and sepsis in the emergency department. Am J Clin Pathol.

[5] Hoeboer SH, van der Geest PJ, Nieboer D, Groeneveld ABJ (2015). The diagnostic accuracy of procalcitonin for bacteraemia: a systematic review and meta-analysis. Clin Microbiol Infect.

[6] de Jong E, van Oers JA, Beishuizen A, Vos P, Vermeijden WJ, Haas LE (2016). Efficacy and safety of procalcitonin guidance in reducing the duration of antibiotic treatment in critically ill patients: a randomised, controlled, open-label trial. Lancet Infect Dis.

[7] Oliveira CF, Botoni FA, Oliveira CRA, Silva CB, Pereira HA, Serufo JC (2013). Procalcitonin versus C-reactive protein for guiding antibiotic therapy in sepsis: a randomized trial. Crit Care Med.

[8] Kip MMA, van Oers JA, Shajiei A, Beishuizen A, Berghuis AMS, Girbes AR (2018). Cost-effectiveness of procalcitonin testing to guide antibiotic treatment duration in critically ill patients: results from a randomised controlled multicentre trial in the Netherlands. Crit Care.

[9] Ratzinger F, Haslacher H, Perkmann T, Pinzan M, Anner P, Makristathis A (2018). Machine learning for fast identification of bacteraemia in SIRS patients treated on standard care wards: a cohort study. Sci Rep.

[10] Parreco JP, Hidalgo AE, Badilla AD, Ilyas O, Rattan R (2018). Predicting central line-associated bloodstream infections and mortality using supervised machine learning. J Crit Care.

[11] Sampson D, Yager TD, Fox B, Shallcross L, McHugh L, Seldon T (2020). Blood transcriptomic discrimination of bacterial and viral infections in the emergency department: a multi-cohort observational validation study. BMC Med.

[12] Roimi M, Neuberger A, Shrot A, Paul M, Geffen Y, Bar-Lavie Y (2020). Early diagnosis of bloodstream infections in the intensive care unit using machine-learning algorithms. Intensive Care Med.

[13] Tsai CM, Lin CHR, Zhang H, Chiu JH, Cheng CY, Yu HR (2020). Using machine learning to predict bacteremia in febrile children presented to the emergency department. Diagnostics (Basel).

[14] Lou AH, Elnenaei MO, Sadek I, Thompson S, Crocker BD, Nassar B (2016). Evaluation of the impact of a total automation system in a large core laboratory on turnaround time. Clin Biochem.

[15] Shao SC, Chan YY, Kao Yang YH, Lin SJ, Hung MJ, Chien RN (2019). The Chang Gung Research Database—a multi-institutional electronic medical records database for real-world epidemiological studies in Taiwan. Pharmacoepidemiol Drug Saf.

[16] Lien F, Wang HY, Lu JJ, Wen YH, Chiueh TS (2021). Predicting 2-day mortality of thrombocytopenic patients based on clinical laboratory data using machine learning. Med Care.

[17] Stijven S, Minnebo W, Vladislavleva E. Separating the wheat from the chaff: on feature selection and feature importance in regression random forests and symbolic regression. In: GECCO ’11: proceedings of the 13th annual conference companion on genetic and evolutionary computation; 2011 Jul 12–16; Dublin, Ireland. New York: Association for Computing Machinery; 2011. p. 623–30.

[18] Uddin S, Khan A, Hossain ME, Moni MA (2019). Comparing different supervised machine learning algorithms for disease prediction. BMC Med Inform Decis Mak.

[19] Youden WJ (1950). Index for rating diagnostic tests. Cancer.

[20] Liu D, Su L, Han G, Yan P, Xie L (2015). Prognostic value of procalcitonin in adult patients with sepsis: a systematic review and meta-analysis. PLoS ONE.

[21] Saito T, Rehmsmeier M (2015). The precision-recall plot is more informative than the ROC plot when evaluating binary classifiers on imbalanced datasets. PLoS ONE.

[22] Taskesen E, Nicorici D, Dang K. Kapla–Meier [Internet]. 2019 [updated 2021 Mar 21; cited 2021 Apr 14]. Available from: https://github.com/erdogant/kaplanmeier.

[23] Schober P, Boer C, Schwarte LA (2018). Correlation coefficients: appropriate use and interpretation. Anesth Analg.

[24] Kim MH, Lim G, Kang SY, Lee WI, Suh JT, Lee HJ (2011). Utility of procalcitonin as an early diagnostic marker of bacteremia in patients with acute fever. Yonsei Med J.

[25] Schuetz P, Mueller B, Trampuz A (2007). Serum procalcitonin for discrimination of blood contamination from bloodstream infection due to coagulase-negative staphylococci. Infection.

[26] Zwang O, Albert RK (2006). Analysis of strategies to improve cost effectiveness of blood cultures. J Hosp Med.

[27] Hu L, Shi QP, Shi M, Liu RX, Wang C (2017). Diagnostic value of PCT and CRP for detecting serious bacterial infections in patients with fever of unknown origin: a systematic review and meta-analysis. Appl Immunohistochem.

[28] Li QQ, Zheng SY, Zhou PY, Xiao ZZ, Wang RL, Li J (2021). The diagnostic accuracy of procalcitonin in infectious patients after cardiac surgery: a systematic review and meta-analysis. J Cardiovasc Med.

[29] Paesmans M, Klastersky J, Maertens J, Georgala A, Muanza F, Aoun M (2011). Predicting febrile neutropenic patients at low risk using the MASCC score: does bacteremia matter?. Support Care Cancer.

[30] Luo X, Zhou W, Yan X, Guo T, Wang B, Xia H (2020). Prognostic value of C-reactive protein in patients with COVID-19. Clin Infect Dis.

[31] Chung H, Lee JH, Jo YH, Hwang JE, Kim J (2019). Circulating monocyte counts and its impact on outcomes in patients with severe sepsis including septic shock. Shock.

[32] Wang JL, Lu XY, Xu XH, Zhang KJ, Gong H, Lv D (2019). Predictive role of monocyte-to-lymphocyte ratio in patients with *Klebsiella pneumonia* infection: a single-center experience. Medicine (Baltimore).

[33] Surana NK, Kasper DL, Jameson JL, Fauci AS, Kasper DL, Hauser SL, Longo DL, Loscalzo J (2018). The human microbiome. Harrison’s principles of internal medicine.

