# Supplementary material for: Bacteremia detection from complete blood count and differential leukocyte count with machine learning: complementary and competitive with C-reactive protein and procalcitonin tests
Source: BMC Infect Dis. 2022 Mar 26;22:287. doi: 10.1186/s12879-022-07223-7 (PMC8962279; doi:10.1186/s12879-022-07223-7)
Supplement: Supplementary file 2 — Additional file 2: Table S2. The AUC performance of different methods with solely CBC/DC data. Table S3. Missing data percentages of biochemistry panels. Table S4. Incorporating biochemistry data compared to CBC/DC only in cases without missing values using random forest. [file 12879_2022_7223_MOESM2_ESM.docx]

**Table S2** The AUC^a^ performance of different methods with solely CBC/DC^b^ data

|  | Random forest | Logistic regression | ANNs^c^ |
| --- | --- | --- | --- |
| 2014  2015  2016  2017  2018  Testing in 2019 | 0.775  0.795  0.794  0.797  0.798  0.802 | 0.750  0.766  0.763  0.770  0.768  0.772 | 0.775  0.799  0.793  0.802  0.806  0.808 |

^a^Area under the ROC curve

^b^Complete blood count / Differential leukocyte count

^c^Artificial neural networks

**Table S3** Missing data percentages of biochemistry panels

| BUN^a^  Cr^b^  Na^c^  K^d^  ALT^e^ | 41.5%  14.8%  24.6%  24.5%  38.1% |
| --- | --- |

^a^Blood urea nitrogen

^b^Creatinine

^c^Sodium

^d^Potassium

^e^Alanine aminotransferase

**Table S4** Incorporating biochemistry data compared to CBC/DC^a^ only in cases without missing values using random forest

|  | Solely CBC/DC  (model from current study) | CBC/DC&CRP^b^+Biochem  (retrained on group without missing values) |
| --- | --- | --- |
| 2014  2015  2016  2017  2018  Testing in 2019 | 0.764  0.775  0.781  0.778  0.777  0.792 | 0.752  0.767  0.768  0.768  0.766  0.780 |

^a^Complete blood count / Differential leukocyte count

^b^Complete blood count / Differential leukocyte count and C-reactive protein
