# Supplementary material for: Bacteremia detection from complete blood count and differential leukocyte count with machine learning: complementary and competitive with C-reactive protein and procalcitonin tests
Source: BMC Infect Dis. 2022 Mar 26;22:287. doi: 10.1186/s12879-022-07223-7 (PMC8962279; doi:10.1186/s12879-022-07223-7)
Supplement: Supplementary file 3 — Additional file 3: Figure S1. a AUC performance of random forest model trained with CBC/DC for predicting infection in CBC/DC group. b PRAUC performance of random forest model trained with CBC/DC for predicting infection in CBC/DC group. Figure S2. a AUC performance of using procalcitonin as the only marker for predicting infection in PCT&CBC/DC group. b PRAUC performance of using procalcitonin as the only marker for predicting infection in PCT&CBC/DC group. Figure S3. a AUC performance of random forest model trained with CBC/DC for predicting infection in PCT&CBC/DC group. b PRAUC performance of random forest model trained with CBC/DC for predicting infection in PCT&CBC/DC group. [file 12879_2022_7223_MOESM3_ESM.docx]

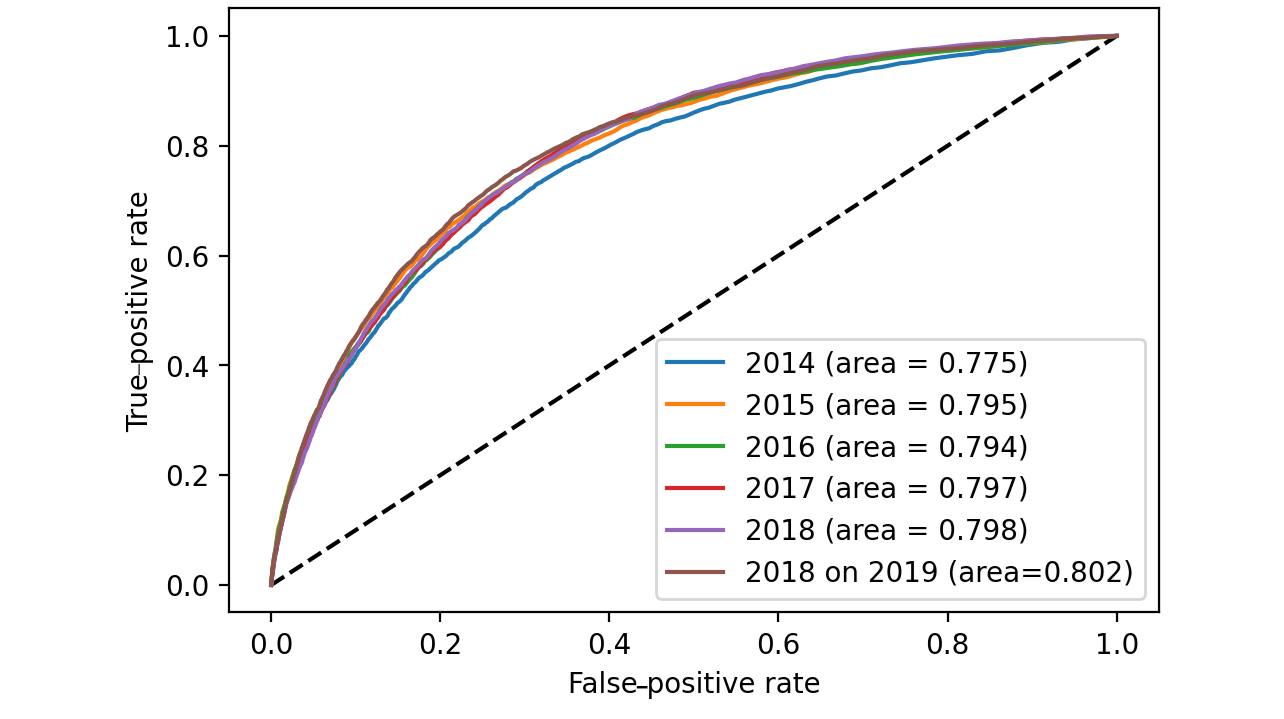


**Fig. S1a** AUC performance of random forest model trained with CBC/DC for predicting infection in CBC/DC group


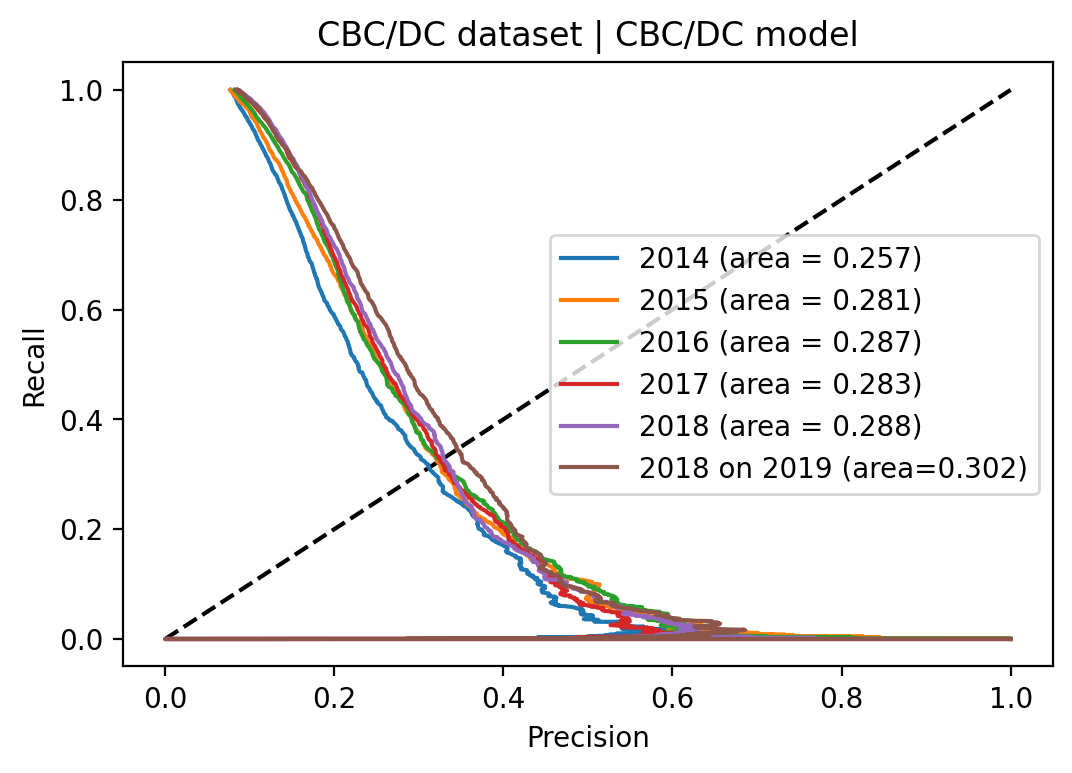


**Fig. S1b** PRAUC performance of random forest model trained with CBC/DC for predicting infection in CBC/DC group


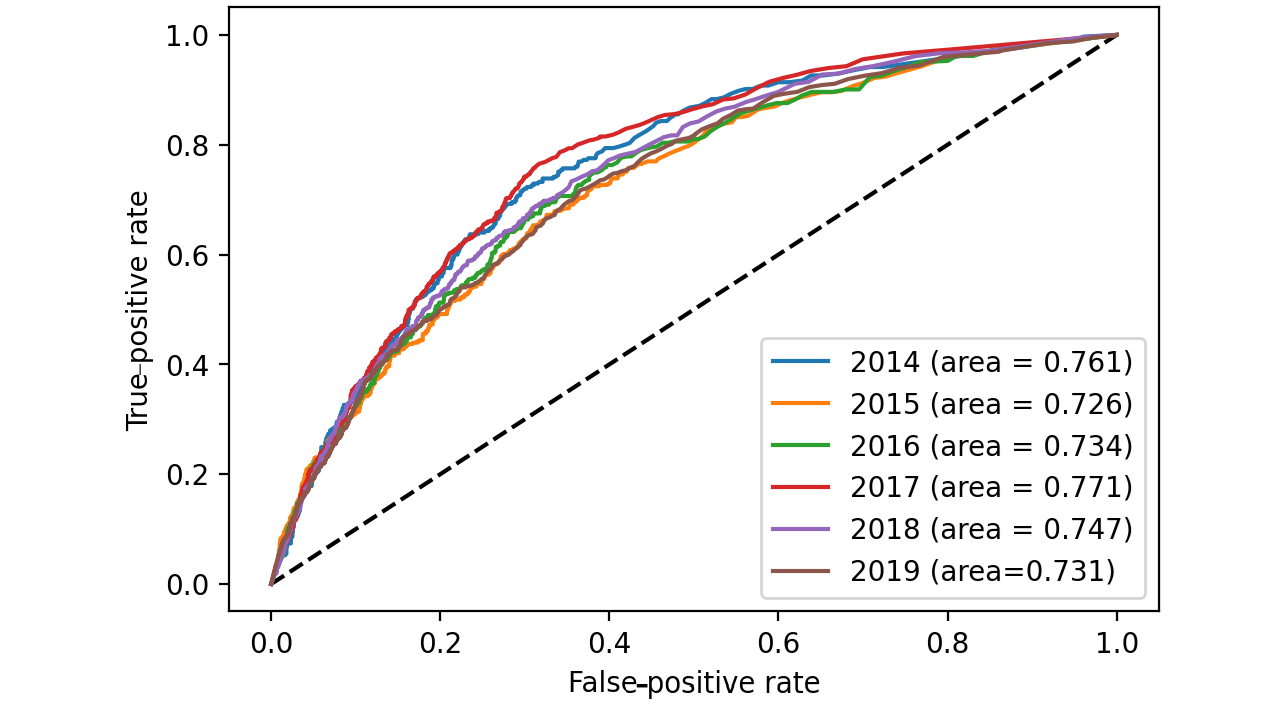


**Fig. S2a** AUC performance of using procalcitonin as the only marker for predicting infection in PCT&CBC/DC group


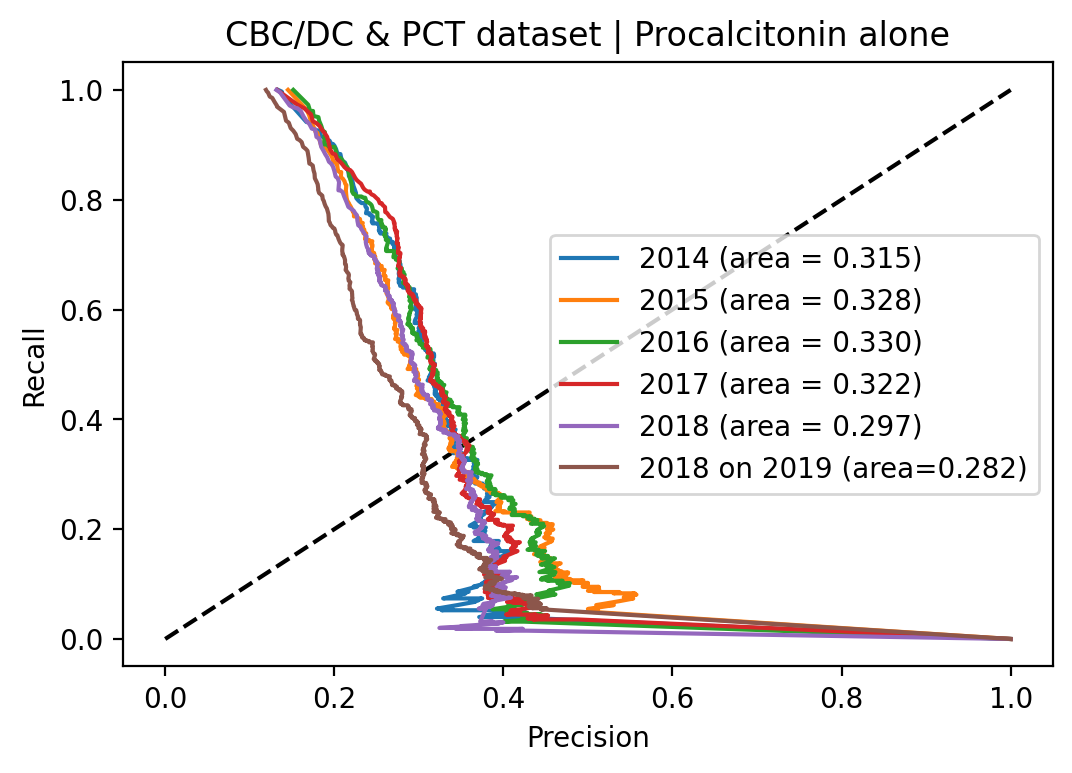


**Fig. S2b** PRAUC performance of using procalcitonin as the only marker for predicting infection in PCT&CBC/DC group


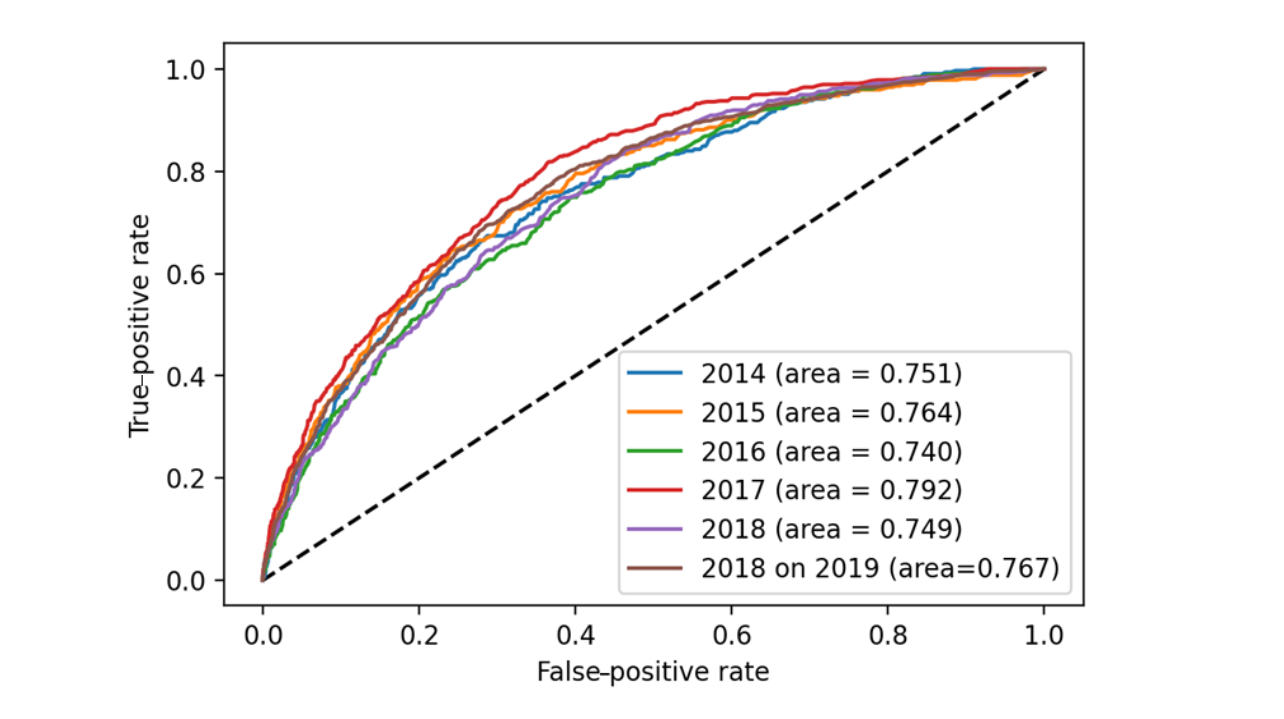


**Fig. S3a** AUC performance of random forest model trained with CBC/DC for predicting infection in PCT&CBC/DC group


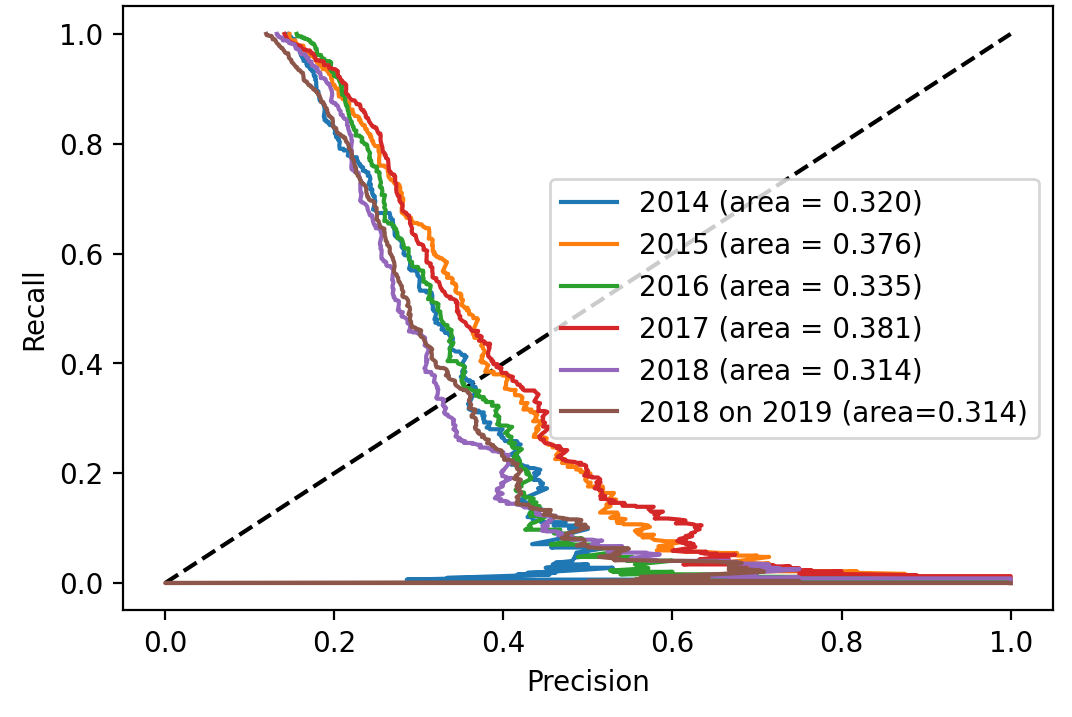


**Fig. S3b** PRAUC performance of random forest model trained with CBC/DC for predicting infection in PCT&CBC/DC group
